# Upholding Scientific Duty Amidst Poisonous Disinformation

**DOI:** 10.7759/cureus.9339

**Published:** 2020-07-22

**Authors:** Daniel A. N. Barbosa, Ricardo De Oliveira-Souza, Alessandra Gorgulho, Antonio De Salles

**Affiliations:** 1 Neurosurgery, Stanford University School of Medicine, Stanford, USA; 2 Specialized Medicine, The Federal University of the State of Rio de Janeiro, Rio de Janeiro, BRA; 3 Neurosurgery, Rede D'or, Sao Paulo, BRA; 4 Neurosurgery, University of California, Los Angeles, USA

**Keywords:** bias identification, use of evidence in policy making, lockdown, brazil

## Abstract

Because of a recent politically-biased *Lancet *editorial, the world’s opinion has been directed against the Brazilian government over the rising numbers of COVID-19 cases in the country. This is an example of reporting data without accounting for important covariates. Epidemiological figures should always be corrected for population size. In fact, Brazil is not even on the list of the 10 countries with the highest number of deaths per 100,000 people. Belgium, the United Kingdom, and Spain are the most affected countries in this regard. The *disinformation *presented by a renowned medical journal has ignited severe criticisms against a Chief-of-State for not promoting a generalized lockdown in a country of continental size. As scientists, we have a duty to stress the caveats of science instead of fueling political attacks, and we should refrain from jumping to uninformed conclusions without considering well-analyzed data. Moreover, while there is no evidence to endorse the efficacy of a generalized lockdown in socioeconomically vulnerable populations, it is undoubtedly associated with severe nationwide adverse effects.

## Editorial

In a recent *Lancet *editorial, the world’s attention was directed toward the rising numbers of confirmed COVID-19 cases and deaths in Brazil [1]. It highlighted some worrisome projections from studies by the Imperial College, London [1]. During the current global health crisis, articles in renowned medical journals have guided public policies and investments in healthcare [2]. Consequently, scientific *information *has* *gained the power to alleviate the suffering of societies; however, *disinformation, *on the other hand,* *causes suffering and ruins livelihoods [3].

Brazil is now the country with the second-highest total number of confirmed COVID-19 cases and deaths [4]. Yet, as scientists, we cannot interpret this piece of raw data without accounting for covariates. Brazil has the sixth largest population in the world; therefore, this raw number must be corrected by its population size. It turns out that Brazil has never been among the 10 large countries with the highest number of deaths per 100,000 people. That list is headed by Belgium, the United Kingdom, and Spain [4]. As of July 12, 2020, Brazil occupies the 12th spot in that list (Table 1). Such a spurious manner of reporting public health data is completely unacceptable even by the lay media, let alone by a renowned scientific journal [1,2,4].

**Table 1:** Coronavirus Resource Center: Cases and Mortality by Country [4] Updated on Sunday, July 12, 2020, at 03:00 AM EDT *Countries with <100,000 inhabitants are not shown

Country*	Confirmed Cases	Deaths	Deaths/100,000 Population
Belgium	62,469	9,782	85.64
United Kingdom	290,504	44,883	67.50
Spain	253,908	28,403	60.79
Italy	242,827	34,945	57.83
Sweden	74,898	5,526	54.27
France	208,015	30,007	44.80
USA	3,245,925	134,777	41.20
Chile	312,029	6,881	36.74
Peru	322,710	11,682	36.52
Ireland	25,611	1,746	35.97
Netherlands	51,136	6,156	35.73
Brazil	1,839,850	71,469	34.12
Ecuador	67,209	5,031	29.45
Mexico	295,268	34,730	27.52
Canada	109,150	8,818	23.79
Switzerland	32,817	1,968	23.11
Panama	44,332	893	21.38
Armenia	31,392	559	18.94

The Lancet focused on politics instead of a scientific analysis of the pandemic's status in Brazil, an odd choice for a scientific publication. Data not corrected for population size was used to build the claim that “*perhaps the biggest threat to Brazil’s Covid-19 response is its president*”, and that “*Brazil’s leadership has lost its moral compass, if it ever had one*” [1]. The editorial failed to provide scientific support, but rather echoed politically-biased ideas. *The Lancet*’s manifesto is merely a science-fueled attack against a Chief-of-State for advising state governors to reopen the economy, and serves instead to encourage a policy of generalized lockdown, which is associated with severe nationwide adverse effects, i.e., increased poverty, conjugal crises, street criminality, depression, suicide, and substance abuse. Calling the lockdown a “*sensible measure*” contrasts with fair criticisms against leaders embracing policies without sufficient evidence of their efficacy and safety. In fact, the universal lockdown adopted by Brazilian mayors and state governors has not slowed down the growing raw number of COVID-19 cases; instead, it has proven to be extremely harmful to several underserved communities [5]. Indeed, physical distancing and hygiene recommendations are impossible to follow in Brazil’s underserved communities, as pointed out by the same editorial [1]. The forceful implementation of these measures has oppressed vulnerable populations, by instilling hunger and crime [5].

It is irresponsible to use poorly analyzed data to accuse leaders, countries, and doctors working with the best of intentions to fight a pandemic that has caused immense misery even in the most developed countries. The use of *disinformation *to point fingers against those fighting in extremely difficult conditions against the same misery, COVID-19, for the benefit of their underserved people is inhuman and unfair, and the heroes of this pandemic deserve better. *The Lancet*’s Editorial Board should at least publish a note apologizing for these unfounded accusations and lack of sensibility [1,2,4]. While we seek to serve the people with a scientific outlook and approaches, a pertinent question arises: *When are our colleagues in the medical field going to stop delivering politically-biased disinformation?*

## References

[1] Editorial (anonymous) (2020). COVID-19 in Brazil: “So what?”. Lancet.

[2] de Oliveira Andrade R (2020). Covid-19: Brazil now has third highest number of cases behind US and Russia. BMJ.

[3] Pacepa IM, Rychlak RJ (2013). Disinformation: Former Spy Chief Reveals Secret Strategies for Undermining Freedom, Attacking Religion, and Promoting Terrorism.

[4] (2020). Johns Hopkins University: Coronavirus Resource Center. http://coronavirus.jhu.edu/data/mortality.

[5] (2020). Lockdown puts Brazilian lives at risk. http://www.wsj.com/articles/lockdown-puts-brazilian-lives-at-risk-11589743232.

